# Context-Dependent Cell Cycle Checkpoint Abrogation by a Novel Kinase Inhibitor

**DOI:** 10.1371/journal.pone.0013123

**Published:** 2010-10-18

**Authors:** Andrew J. Massey, Jenifer Borgognoni, Carol Bentley, Nicolas Foloppe, Andrea Fiumana, Lee Walmsley

**Affiliations:** Vernalis (R&D) Ltd., Granta Park, Cambridge, United Kingdom; National Cancer Institute, United States of America

## Abstract

**Background:**

Checkpoint kinase 1 and 2 (Chk1/Chk2), and the Aurora kinases play a critical role in the activation of the DNA damage response and mitotic spindle checkpoints. We have identified a novel inhibitor of these kinases and utilized this molecule to probe the functional interplay between these two checkpoints.

**Principal Findings:**

Fragment screening, structure guided design, and kinase cross screening resulted in the identification of a novel, potent small molecule kinase inhibitor (VER-150548) of Chk1 and Chk2 kinases with IC_50_s of 35 and 34 nM as well as the Aurora A and Aurora B kinases with IC_50_s of 101 and 38 nM. The structural rationale for this kinase specificity could be clearly elucidated through the X-ray crystal structure. In human carcinoma cells, VER-150548 induced reduplication and the accumulation of cells with >4N DNA content, inhibited histone H3 phosphorylation and ultimately gave way to cell death after 120 hour exposure; a phenotype consistent with cellular Aurora inhibition. In the presence of DNA damage induced by cytotoxic chemotherapeutic drugs, VER-150548 abrogated DNA damage induced cell cycle checkpoints. Abrogation of these checkpoints correlated with increased DNA damage and rapid cell death in p53 defective HT29 cells. In the presence of DNA damage, reduplication could not be observed. These observations are consistent with the Chk1 and Chk2 inhibitory activity of this molecule.

**Conclusions:**

In the presence of DNA damage, we suggest that VER-150548 abrogates the DNA damage induced checkpoints forcing cells to undergo a lethal mitosis. The timing of this premature cell death induced by Chk1 inhibition negates Aurora inhibition thereby preventing re-entry into the cell cycle and subsequent DNA reduplication. This novel kinase inhibitor therefore serves as a useful chemical probe to further understand the temporal relationship between cell cycle checkpoint pathways, chemotherapeutic agent induced DNA damage and cell death.

## Introduction

Cell cycle checkpoints protect the fidelity of DNA replication and division and ensure the correct ordering of cell cycle events. Once the information encoded in DNA is lost, it cannot be replaced, therefore these pathways are vital for maintaining genomic integrity and preventing carcinogenesis [1]. There are several checkpoints regulating cell cycle progression: those that are activated during the G1-, S- or G2-phase of the cell cycle in response to DNA damage. This DNA damage can arise either as a result of endogenous stimuli or through external mechanisms (including genotoxic stress or chemotherapeutic drugs). In addition, a second kind of checkpoint, here termed the mitotic spindle checkpoint, is activated during every cell cycle and only silenced once all chromosomes are properly attached to a bipolar spindle and ensures accurate chromosome segregation and protects against aneuploidy.

DNA damaging agents, such as cisplatin, carboplatin, irinotecan and doxorubicin, along with ionizing radiation are the mainstays of cancer therapy. While they have different mechanisms of action, they all directly or indirectly induce DNA damage thereby activating DNA damage checkpoints and induce cell cycle arrest in G1, S, or at the G2-M transition. In mammalian cells, the key effector proteins are p53 and the checkpoint kinases Chk1 and Chk2. A large proportion of human cancers exhibit dysregulation of p53 function (e.g. by p53 mutation or loss of p53 expression) and therefore are unable to activate transcription of the CDK inhibitor, p21, which is required for arrest in G1. These human tumors are thought to be highly reliant on the Chk kinases to protect them in response to DNA damaging insults [2]–[4]. Chk1 is required for the signal evoked by damaged DNA to prevent entry into mitosis; it is widely assumed that Chk1 inhibitors kill cells by overriding this constraint allowing entry into a lethal mitosis.

Damage sensors that recognize double strand breaks or protein complexes that recognize replication stress activate the transducing kinases ATM and ATR. In turn, these kinases directly activate the effector kinases Chk1 and Chk2. Chk1 and Chk2 negatively regulate the Cdc25 family of phosphatases thereby preventing cell cycle progression as well as directly modulating repair proteins resulting in increased lesion repair. Chk1 appears to be the crucial effector kinase as both biochemical and genetic studies have demonstrated it to be indispensible for the S and G2/M checkpoints [5]. Chk1 inhibition, therefore, represents a novel therapeutic strategy to increase the lethality of DNA-damaging chemotherapeutic drugs in p53 pathway defective cancers. Abrogation of the remaining intact checkpoint should result in increased tumor cell death. This “synthetic lethality” approach should increase the therapeutic index of chemotherapeutic drug as normal cells remain protected by their functional p53 pathway. This approach has started to be tested clinically with small molecule inhibitors of Chk1 (AZD7762, PF-477736, XL844 and SCH900776) currently undergoing Phase I clinical evaluation in combination with gemcitabine, irinotecan and cytarabine [6]–[9]. Recent work has suggested that Chk1 may also be required for the normal operation of the spindle assembly checkpoint [10], which may account for the ability of the Chk1 inhibitor PF-477736 to potentiate the efficacy of docetaxel in xenografts [11].

Spindle checkpoint function and thus accurate mitosis relies on the Mad proteins Mad1, Mad2 and BubR1, the Bub proteins Bub1 and Bub3, the mitotic kinases Aurora A and Aurora B, as well as Chk1 [10], [12]. Several antimitotic drugs including the taxanes and the *vinca* alkaloids, via their effects on microtubules, prevent the formation of a normal mitotic spindle, resulting in spindle checkpoint activation. These agents impose mitotic arrest, usually leading to apoptosis either in mitosis or, more often, in the post-mitotic G1-phase following mitotic escape [13].

The Aurora family of Ser/Thr kinases consists of three members designated Aurora A, B and C, all of which play a role in mitotic progression [14], [15]. All three Aurora kinases are implicated in cancer development and progression, and their overexpression is common in a wide variety of human cancers [16], [17]. Aurora kinases have become popular targets for cancer drug discovery with at least thirteen small molecule inhibitors currently in Phase I and II clinical evaluation [18], [19]. Two of the molecules that have demonstrated the potential of this approach are VX680 (MK-0457, Vertex/Merck) and AZD1152 (AstraZeneca). Inhibiting Aurora B results in premature exit from mitosis, failed cytokinesis followed by induction of reduplication (that is the formation of cells with a >4N DNA content). Histone H3 phosphorylation is a widely used biomarker of Aurora B activity [20]. Through their ability to induce mitotic checkpoint malfunction, Aurora kinase inhibitors synergize with agents that target the mitotic spindle, such as paclitaxel and nocodazole [21], [22].

Using fragment screening, structure guided drug design and kinase cross screening we have identified VER-150548, a potent small molecule inhibitor of both Chk1 and Chk2, and Aurora A and Aurora B kinases. Here we demonstrate that in unperturbed cells, VER-150548 induced a cellular phenotype consistent with Aurora kinase inhibition but in the presence of DNA damage, a Chk1 inhibitor phenotype. We have therefore utilised VER-150548 as a useful chemical probe to further understand the interplay between these two signalling pathways and the temporal factor that determines the predominant cellular signalling pathway.

## Results and Discussion

### VER-150548 Inhibits Chk and Aurora kinases

The benzimidazole pyrazoles were originally identified from fragment screening and elaborated, using structure guided drug design, as small molecule inhibitors of protein kinases. VER-150548 (Figure S1) is a potent lead inhibitor of checkpoint kinases and inhibited Chk1 and Chk2 with IC_50_s of 35 and 34 nM. Kinase cross screening identified VER-150548 as a potent inhibitor of Aurora A (IC_50_ 101 nM) and Aurora B (IC_50_ 38 nM) kinases (Table 1). VER-150548 was more than 50-fold selective against CDK1 and 2. In a larger panel of 45 kinases, VER-150548 at 1 µM inhibited cTAK1, Src and Syk >70% and AMPK, CAMK2, CAMK4, FLT3, MARK1, MST2, PDPK1 and RSK1 >90%. VER-150548 at 1 µM did not inhibit MAPKAPK2, a kinase implicated as important in DNA damage response checkpoints.

**Table 1:** Structure and *in vitro* activity of VER-150548.

	**Kinase**					
	AurA	AurB	Chk1	Chk2	CDK1	CDK2
IC_50_ (nM)	101	38	35	34	2800	8000
	**HCT116**		**HT29**		**MDA-MB-468**	
	72 h	120 h	72 h	120 h	72 h	120 h
GI_50_ (µM)	3.4	0.21	4.6	0.42	0.38	0.30

Values are the average of at least two independent determinations.

### Structural rationale for the inhibition of Chk1 and Aurora kinases by VER-150548

**Figure 1:**
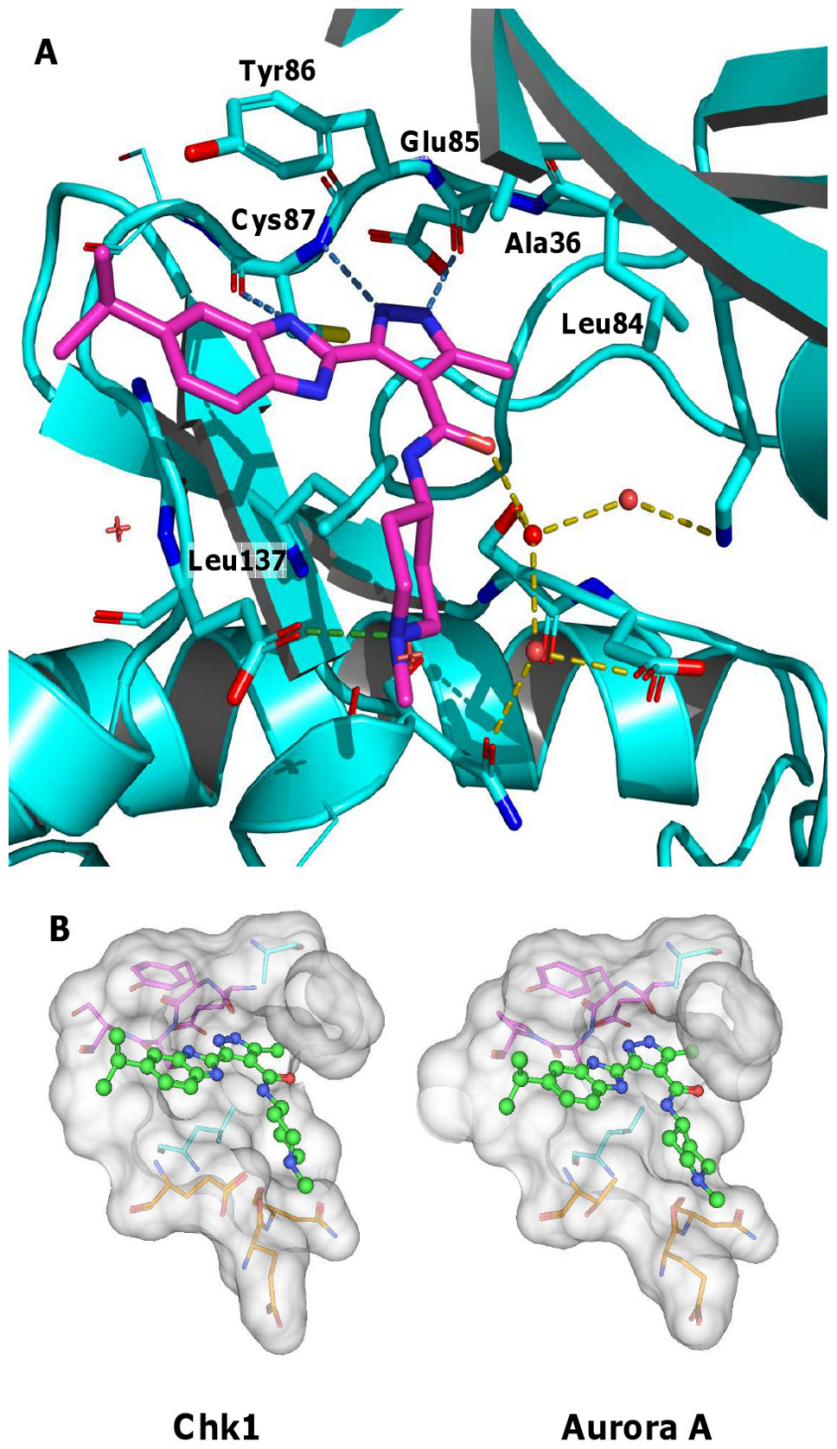
Structural rationale for inhibitory activity of VER-150548. (A) X-ray structure of VER-150548 bound to Chk1 highlighting key molecule-protein interactions. Only the protein binding site in the direct vicinity of VER-150548 is shown and the glycine loop has been hidden for clarity. (B) X-ray crystal structure of VER-150548 (ball and stick with green carbon atoms) binding to the ATP-binding site of Chk1 (left) or docking binding mode of Aurora A (right). Selected residues are highlighted: the structurally conserved kinase hinge residues in magenta, two conserved residues which form apolar contacts above (alanine) and below (leucine) the aromatic core of VER-150548 in light blue and the polar residues (orange) lining the solvent exposed “sugar-phosphate” pocket.

The structure of the Chk1-VER-150548 complex was obtained by X-ray crystallography at high resolution (1.9 Å), confirming that VER-150548 inhibits Chk1 by occupying its ATP-binding site (Figure 1A and B). The Chk1 kinase hinge motif (Glu85-Tyr86-Cys87) hydrogen-bonds the pyrazolo-benzimidazole moiety of VER-150548, following a pattern typical of kinase-inhibitor complexes. The pyrazolo-benzimidazole core is deeply buried in the Chk1 binding site with an excellent overall shape complementarity fit. The core of VER-150548 is sandwiched between apolar side-chains Ala36 and Leu137, and the methyl substituent of the pyrazole fits snugly against the gatekeeper residue (Leu84). Burial of this apolar surface area is expected to be a major contributing factor to the potency of VER-150548 for Chk1. The piperidine of VER-150548 binds in the solvent exposed periphery of the binding site, which would normally accommodate the sugar-phosphate of ATP. Thus, the structural elements underpinning the binding of VER-150548 to Chk1 are precisely defined, allowing the modeling with high confidence of the binding mode in similar kinase ATP-binding sites. The binding mode of VER-150548 in Aurora A was modeled based on the crystal structures of close analogues of VER-150548 bound to this kinase allowing the placing of VER-150548 with a high degree of certainty. There is an overall shape similarity between the ATP-binding sites of Chk1 and Aurora A (Figure 1B). Many conserved structural features exist between these two binding clefts, including the geometry of the kinase hinge backbone, the same gatekeeper side-chain (leucine), several apolar residues contacting the pyrazolo-benzimidazole core, and the more polar character of the peripheral pocket accommodating the piperidine moiety. The overall complementarity of shape and chemical interactions observed between VER-150548 and Chk1 is largely conserved in Aurora A and is consistent with VER-150548 being a potent inhibitor of these two kinases.

### VER-150548 Inhibits Aurora Kinases in Human Carcinoma Cells

Inhibition of Aurora kinases results in cell death after extended time periods. VER-150548 inhibited the proliferation of human cancer cell lines with GI_50_s in the range 0.38–4.6 µM following 72 hour treatment (Table 1). The potency increased in line with extended incubation times; GI_50_s were in the range 0.21–0.42 µM after 120 hour incubation.

**Figure 2:**
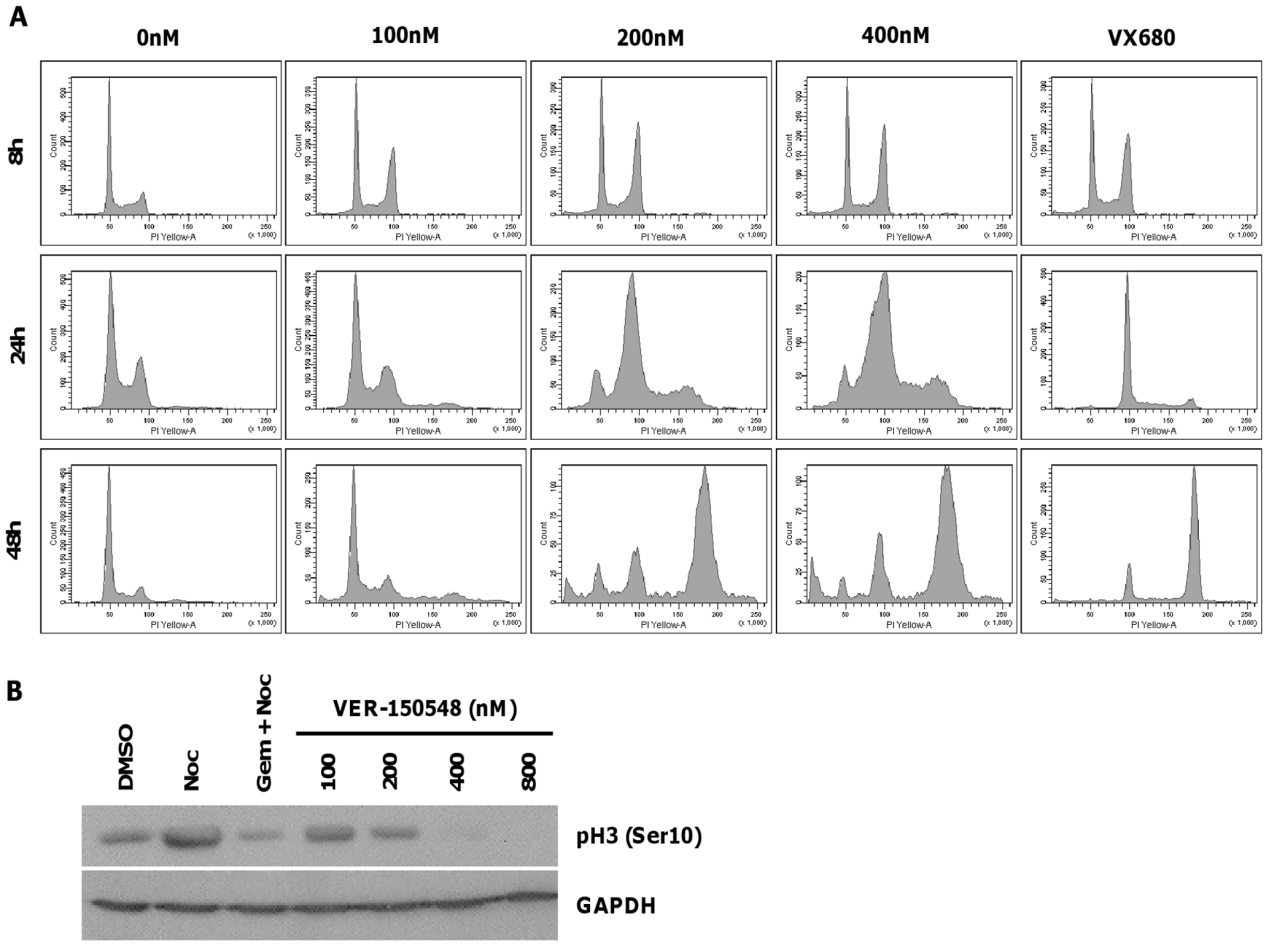
VER-150548 induces polyploidy and inhibits Histone H3 Ser10 phosphorylation in HT29 cells. (A) HT29 cells were exposed to the indicated concentrations of VER-150548 for 8, 24 or 48 hours. Cell cycle profiles were determined by propidium iodide staining on a FACSArray cytometer. (B) HT29 cells were exposed to the indicated concentrations of VER-150548 for 24 hours. Cell lysates were prepared and the level of pH3 (Ser10) determined by western blotting.

siRNA mediated knockdown of Aurora B or addition of Aurora B kinase inhibitors results in failed cytokinesis, which is followed by the onset of DNA replication in cells that already have a 4N DNA content [23]. Flow cytometry was utilized to evaluate the ability of VER-150548 to induce reduplication and inhibit Histone H3 phosphorylation in carcinoma cells. Treatment with 200 nM or greater VER-150548 resulted in accumulation of cells with a 4N DNA content after 8 to 24 hours, which we tentatively attribute to arrest at the G2/M transition following Aurora A inhibition (Figure 2A) [24]. Longer incubations led to a greatly increased number of cells with 8N DNA content indicating that the compound blocked cell division without preventing chromosomal DNA replication. The Aurora kinase inhibitor VX680 [25] similarly caused G2/M arrest at early time points and subsequent reduplication following extended incubation. VER-150548 induced reduplication in HCT116 and MDA-MB-468 cells at concentrations comparable to those that induced reduplication in HT29 cells (data not shown). Aurora B is responsible for most of the kinase activity directed against Histone H3 on serine 10 (pH3 (Ser10)), hence phosphorylation at this site can be employed as a biomarker of Aurora B kinase activity [26]. VER-150548 induced a decrease in pH 3 (Ser10) levels in asynchronous HT29 cells (Figure 2B), though slightly higher concentrations of VER-150548 were required to reduce pH 3 (Ser10) levels than were necessary to induce reduplication.

### VER-150548 Abrogates Cell Cycle Checkpoints

**Figure 3:**
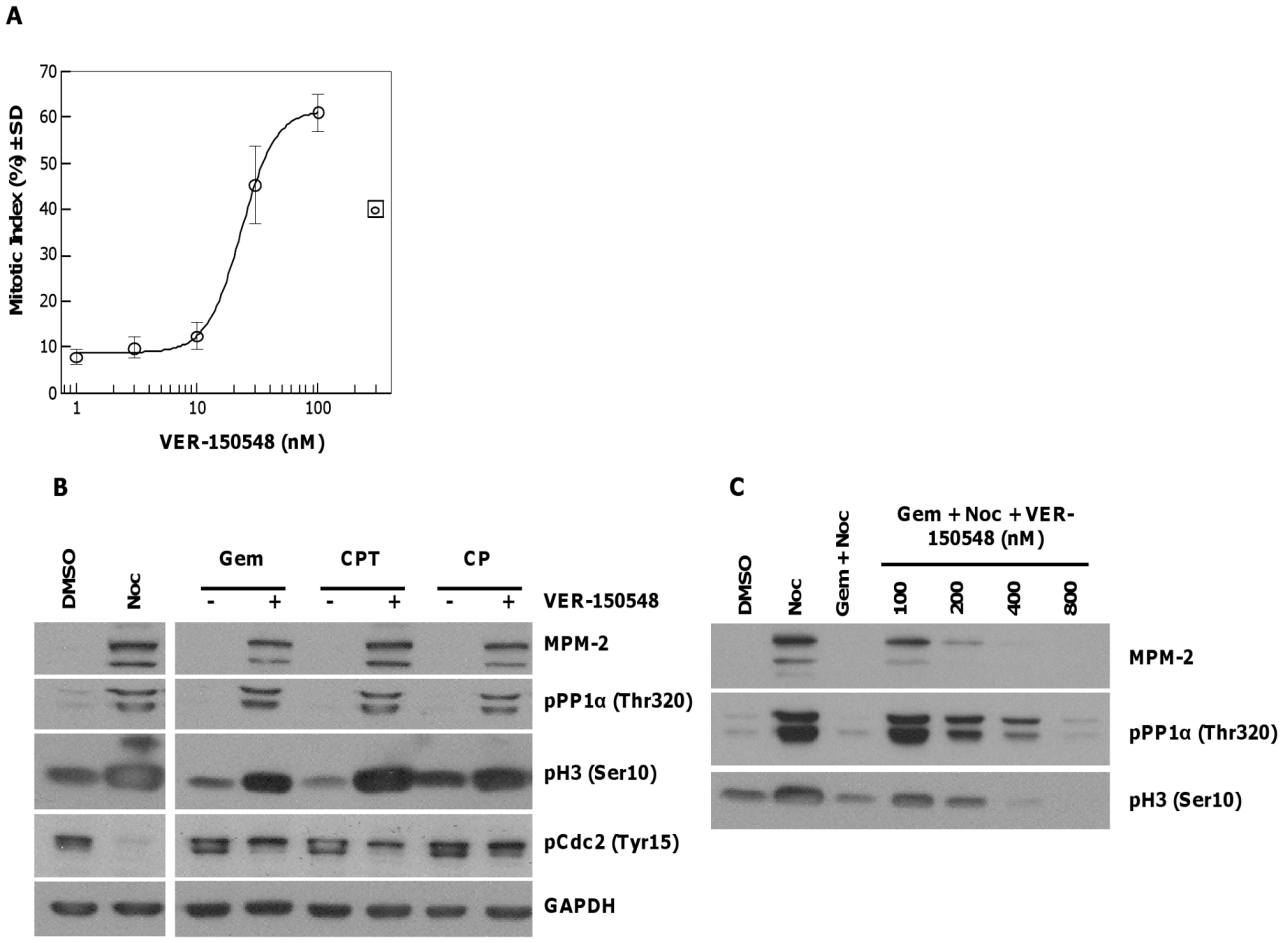
VER-150548 abrogates the DNA damage checkpoint induced by gemcitabine, camptothecin or cisplatin. (A) HT29 cells were exposed to gemcitabine (25 nM) for 16 hours followed by increasing concentrations of VER-150548 in the presence of nocodazole for 24 hours. Mitotic cells were determined following staining for pH3 (Ser10) and counterstaining with DAPI. Values are the average of four independent determinations ± SD. (B) HT29 cells were treated with gemcitabine (Gem, 25 nM), camptothecin (CPT, 50 nM) or cisplatin (CP, 12 µM) for 16 hours followed by 0 (-) or 100 nM (+) VER-150548 in the presence of nocodazole for 24 hours. Cell lysates were prepared and analyzed by western blotting. (C) HT29 cells were treated with gemcitabine (Gem, 25 nM), for 16 hours followed by 0 to 800 nM VER-150548 in the presence of nocodazole for 24 hours. Cell lysates were prepared and analyzed by western blotting.

The checkpoint kinase Chk1 is essential for arresting the cell cycle of p53 defective cells in response to DNA damage [5] including that induced by cyototoxic chemotherapeutic drugs such as gemcitabine and cisplatin. The ability of VER-150548 to abrogate gemcitabine induced S-phase arrest was determined in p53-defective HT29 cells. Following treatment with gemcitabine then VER-150548 plus nocodazole, cells were examined for expression of pH 3 (Ser10); a marker indicative of mitosis. Nocodazole arrests cells in mitosis whilst gemcitabine, in combination with nocodazole, results in S-phase arrest with a low proportion of pH 3 (Ser10) positive mitotic cells. VER-150548 abrogated gemcitabine induced S-phase arrest leading to the accumulation of cells in mitosis with an EC_50_ of 23 nM (Figure 3A). Gemcitabine, camptothecin or cisplatin arrested HT29 cells in either S- or G2-phase (as evidenced by high pCdc2 (Tyr15) and low MPM-2, pPP1α (Thr320) and pH 3 (Ser10) levels). This cell cycle arrest could be abrogated by VER-150548, allowing cells to progress through into mitosis and subsequent trapping by nocodazole (Figure 3B). Checkpoint abrogation occurred at concentrations of VER-150548 as low as 100 nM. At higher concentrations (>400 nM), a decrease in mitotic markers was observed reflecting the Aurora kinase inhibitory activity of the molecule (Figure 3C). DNA damage induced checkpoint abrogation appeared reliant on the absence of functional p53 as no checkpoint abrogation was observed in the p53 proficient colon carcinoma cell line HCT116 (Figure S2).

**Figure 4:**
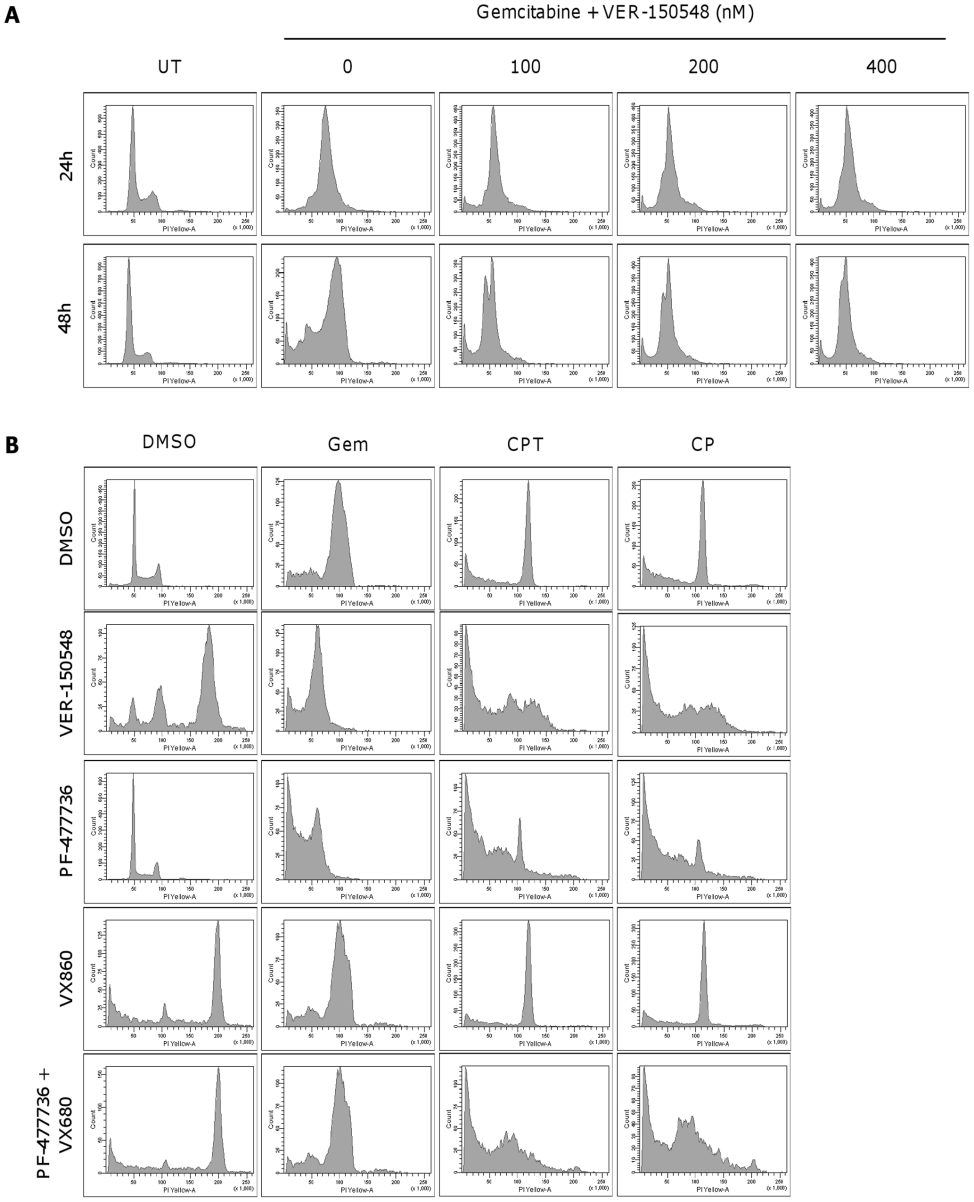
Cell cycle changes induced by checkpoint abrogation by VER-150548. (A) HT29 cells were mock treated with DMSO or exposed to gemcitabine for 16 hours followed by VER-150548 for a further 24 or 48 hours. (B) HT29 cells were mock treated with DMSO or exposed to gemcitabine, camptothecin or cisplatin for 16 hours followed by DMSO, VER-150548 (200 nM), PF-477736 (400 nM), VX680 (400 nM) or a combination of PF-477736 and VX680 for a further 48 hours. Fixed cells were analyzed by flow cytometry.

**Figure 5:**
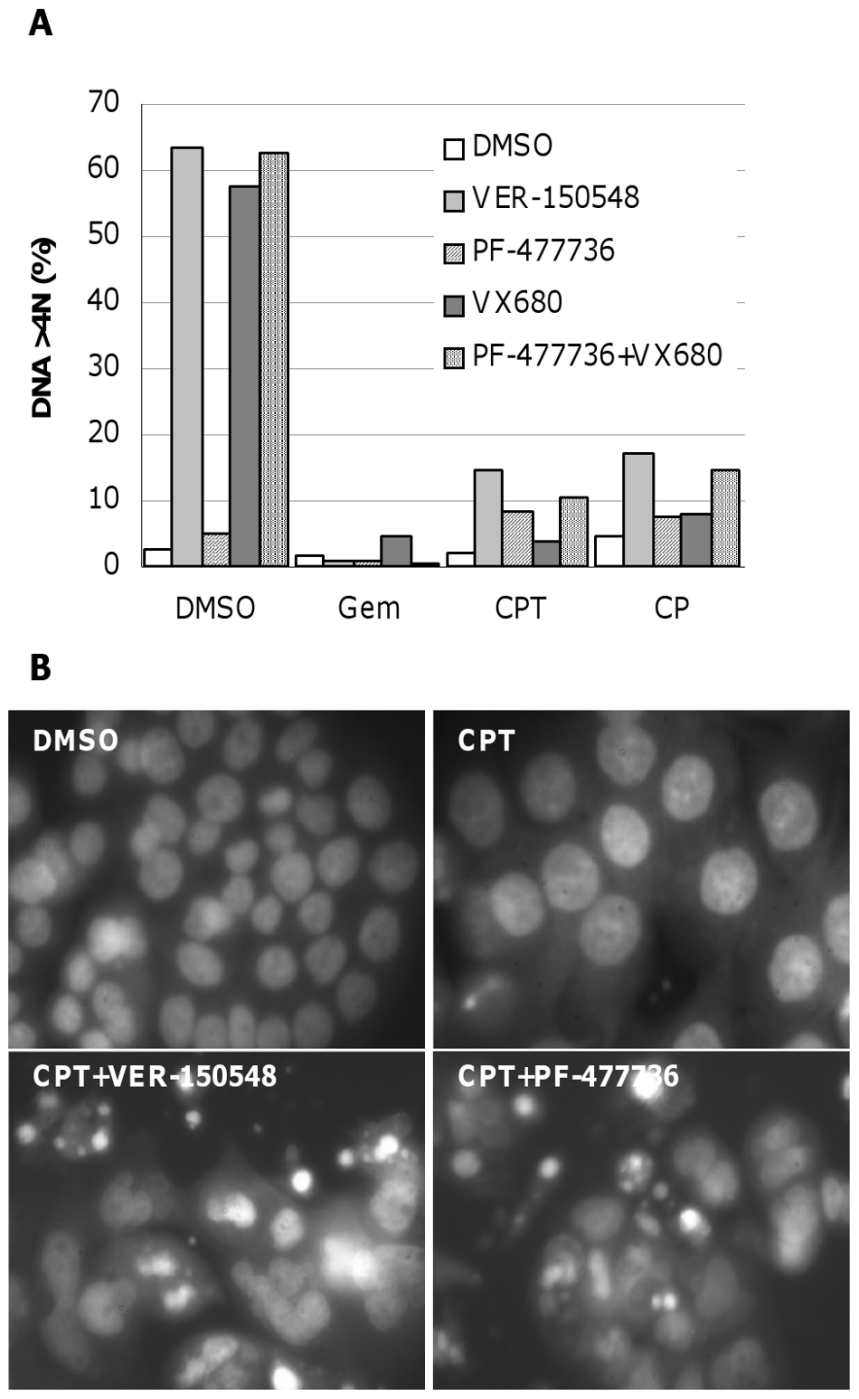
Checkpoint abrogation does not result in reduplication. (A) The fraction of cells with a DNA content >4N was quantitated from the cell cycle profiles in Figure 4. (B) HT29 cells were treated with camptothecin for 16 hours followed by DMSO, VER-150548 (200 nM) or PF-477736 (400 nM) for a further 48 hours. Following fixation with paraformaldehyde, nuclei were stained with Hoescht 33342 and imaged at 400× magnification using a Zeiss Axiovert microscope.

Abrogation of DNA damage induced cell cycle checkpoints by VER-150548 resulted in rapid cell death, as confirmed by the large increase in cells with a DNA content <2N after 24 and 48 hours (Figure 4A). Cell death occurred in a dose and time dependent fashion with the greatest cell death occurring after 48 hours. The Chk1 inhibitor PF-477736 [7] similarly abrogated DNA damage induced cell cycle arrest whilst the Aurora inhibitor VX680 [25] was unable to override the DNA damage induced arrest (Figure 4B). Combination treatment of camptothecin or cisplatin with VER-150548 resulted in a small fraction (<17%) of cells with a DNA content >4N (Figure 4B and 5A). This was noticeably less than those cells treated with VER-150548 alone (63% DNA content >4N). The combination treatment induced a DNA content between 4 and 7N and this did not match the 8N DNA profile expected from reduplication following Aurora inhibition. Similarly, in cells treated with DNA damaging agents followed by PF-477736 plus VX680, only a small percentage had a DNA content >4N (11–15%). Again the DNA content of this fraction of cells varied from 4N to around 7N and did not correspond with the 8N predicted from reduplication. Hoescht nuclear staining of cells treated with camptothecin plus VER-150548 or PF-477736 indicated a high degree of cells with aberrant nuclear morphology indicative of a high degree of chromosomal abnormalities and damage (Figure 5B).

**Figure 6:**
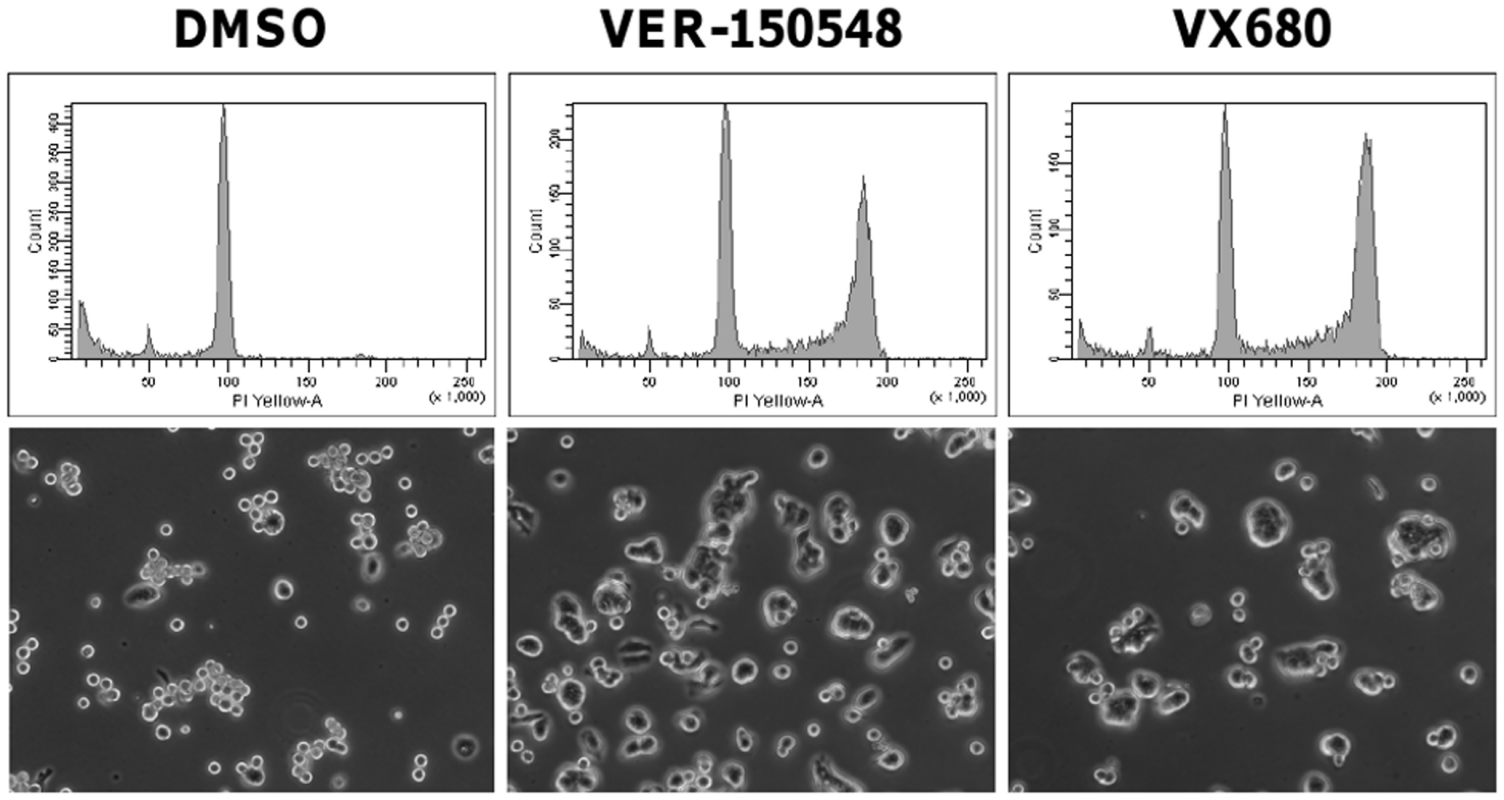
VER-150548 abrogates Paclitaxel induced mitotic arrest. HT29 cells were treated with 100 nM paclitaxel for 18 hours, media removed and treated with VER-150548 (200 nM) or VX680 (400 nM) for a further 24 hours before being either fixed and analyzed by flow cytometry or visualized under a Zeiss Axiovert microscope at 200× magnification.

An additional checkpoint, the spindle assembly checkpoint, monitors the proper alignment of chromosomes during mitosis and can be activated by anti-mitotic drugs such as paclitaxel. Treatment of paclitaxel arrested HT29 cells with VER-150548 or VX680 resulted in spindle checkpoint malfunction and the exiting of cells from mitosis (Figure 6). Twenty four hours after the removal of paclitaxel, 65.4% of cells remained arrested in G2 or M compared to 34.3 and 28.5% treated with 200 nM VER-150548 or 400 nM VX680 respectively. Microscopic analysis of combination treated cells indicated a return to an interphase morphology whilst those treated with DMSO maintained a mitotic morphology (Figure 6).

### Checkpoint Abrogation Potentiates DNA Damage and Cell Death

**Figure 7:**
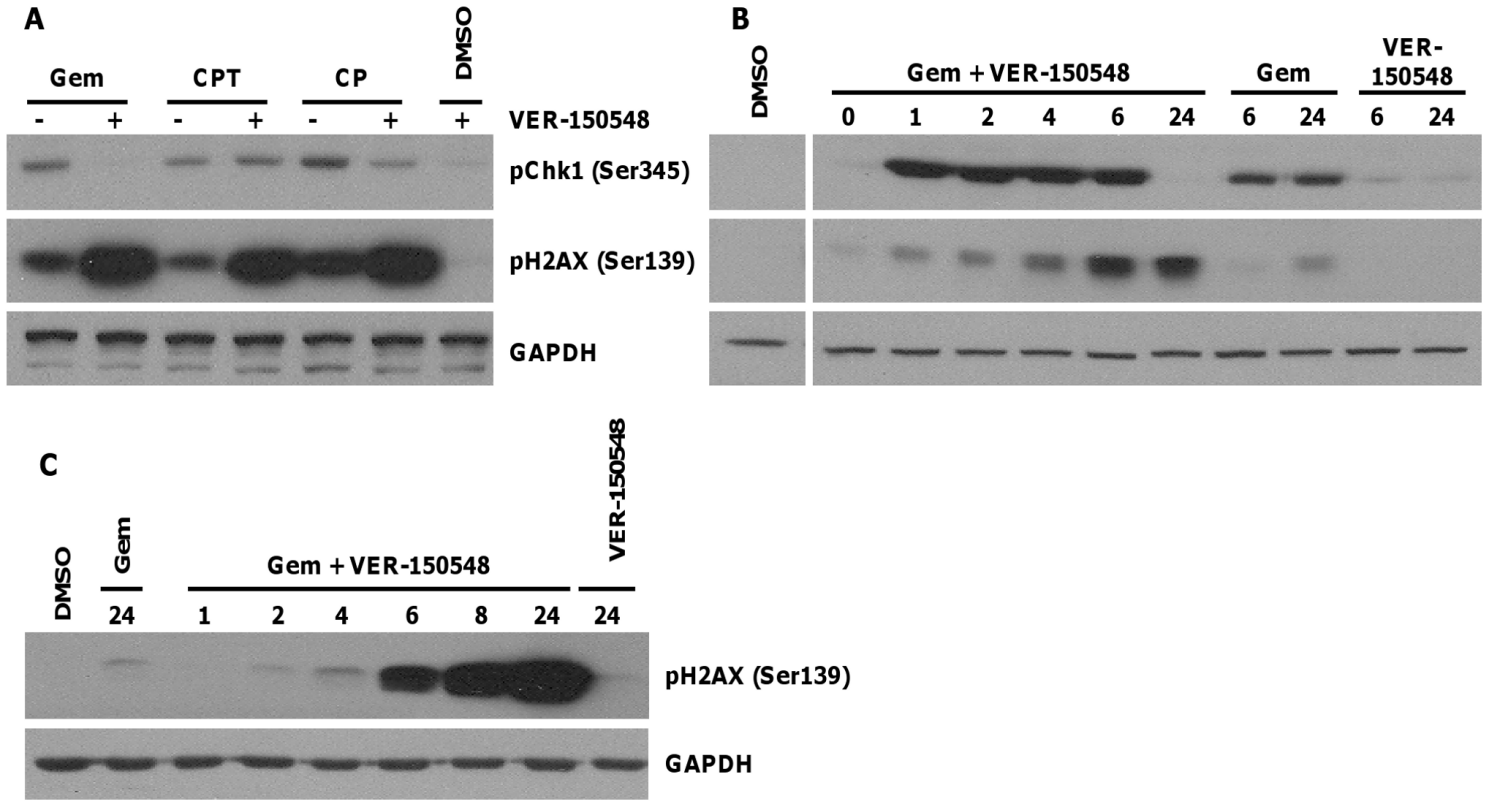
Checkpoint abrogation increases DNA damage. (A) HT29 cells were treated with gemcitabine, camptothecin or cisplatin for 16 hours followed by 0 (-) or 100 nM (+) VER-150548 for 24 hours. (B) HT29 cells were treated with or without gemcitabine for 16 hours followed by VER-150548 (100 nM) for the indicated times. (C) HT29 cells were exposed to gemcitabine followed by VER-150548 (100 nM). At the indicated time points, drug containing media was removed and replaced with drug free. All cells were harvested 24 hours post the addition of VER-150548 and the indicated proteins analyzed by western blotting. All lanes were from the same gel and experiment; irrelevant lanes have been removed for clarity.

Following DNA damage, the histone variant H2AX is phosphorylated on Ser139 by ATM/ATR and forms nuclear foci at the sites of damage thereby serving as a useful marker of cellular levels of DNA damage. Inhibition of checkpoint kinases following cytotoxic chemotherapy results in increased DNA strand breaks due to stalled replication fork collapse and replication of damaged DNA. In addition to phosphorylating H2AX, ATM/ATR also phosphorylates Chk1 at Ser345. Treatment of HT29 cells with gemcitabine, camptothecin or cisplatin for 40 hours increased pChk1 (Ser345) and, to a lesser extent, pH2AX (Ser139). The sequential treatment of HT29 cells with a DNA damaging agent for 16 hours followed by VER-150548 for a further 24 hours resulted in a decrease in pChk1 (Ser345) but a large increase in pH2AX (Ser139) (Figure 7A). Abrogation of gemcitabine induced arrest resulted in the rapid formation of DNA strand breaks as visualized by the phosphorylation of Chk1 at Ser345 within 1 hour and H2AX at Ser139 within 6 hours (Figure 7B). H2AX phosphorylation was maintained up to 24 hours after the addition of VER-150548. In contrast, Chk1 was dephosphorylated after 6 hours resulting in a complete loss of Ser345 phosphorylation by 24 hours. A washout experiment confirmed that 6 hour exposure of VER-150548 was sufficient to induce H2AX phosphorylation and that this was maintained for at least 18 hours after the removal of VER-150548 (Figure 7C).

**Figure 8:**
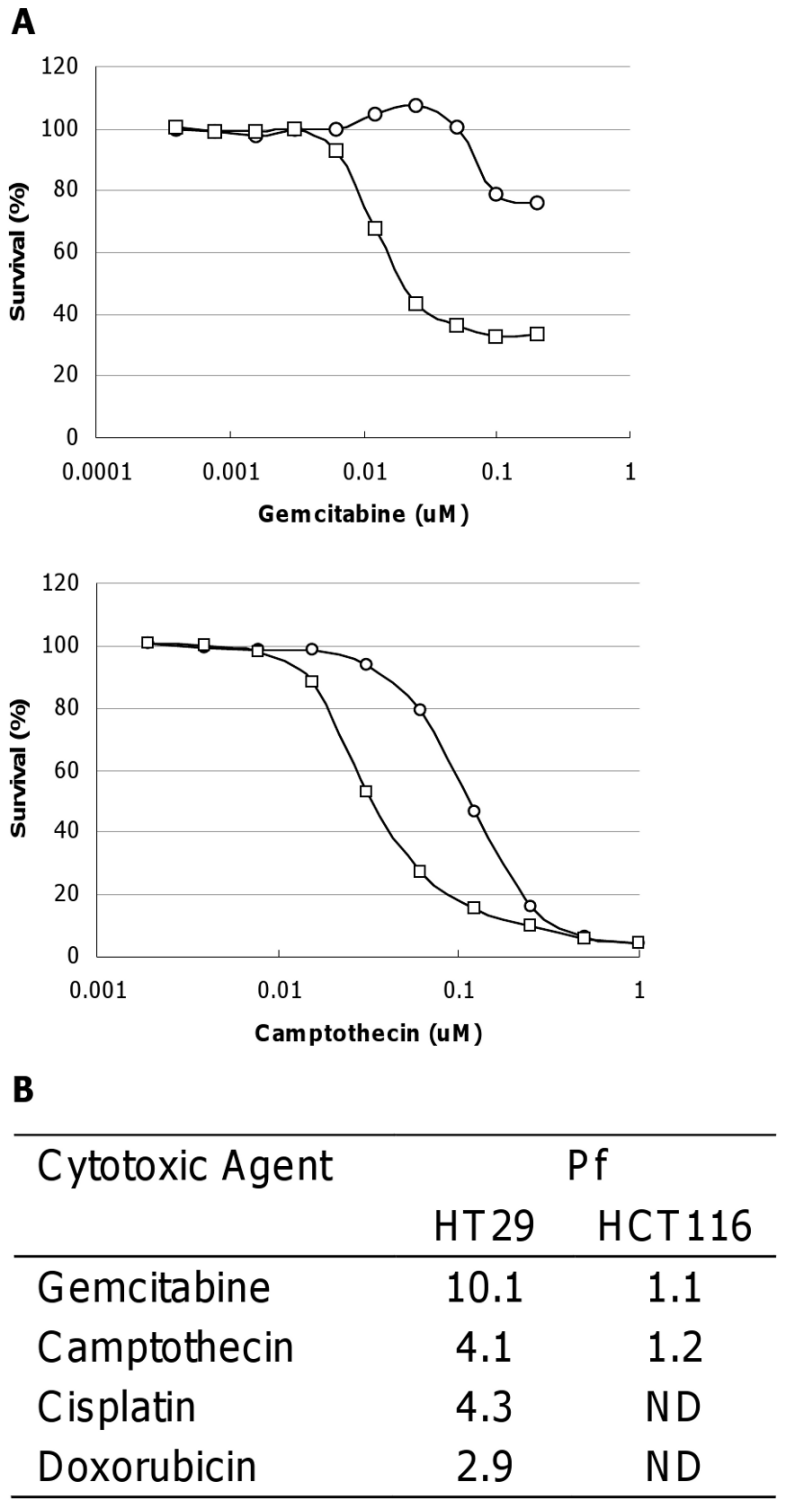
VER-150548 potentiates the cytotoxic activity of chemotherapeutic drugs in HT29 colon carcinoma cells. (A) HT29 cells were co-treated with gemcitabine (top) or camptothecin (bottom) in the absence (circles) or presence (squares) of VER-150548 (100 nM) for 72 hours. Example curves are illustrated. (B) Potentiation factors (P_f_) were calculated as GI_50(cytotoxic agent alone)_/GI_50(combination treatment)_ for VER-150548 in combination with various chemotherapeutic agents. Values are the average of four independent determinations.

Chk1 inhibitors potentiate the growth inhibitory activity of a variety of chemotherapeutic agents in p53 defective cancer cells [6], [7]. VER-150548 potentiated the growth inhibitory activity of gemcitabine, cisplatin, camptothecin and doxorubicin in p53 mutant HT29 cells (Figure 8A and B). The range of concentrations at which VER-150548 enhanced gemcitabine and camptothecin cytotoxicity was substantial: robust potentiation was observed between 50 and 400 nM VER-150548 and correlated closely with increased DNA damage. In common with other Chk inhibitors, the greatest potentiation was observed when VER-150548 was combined with gemcitabine [6], [7]. As expected, this potentiation was dependent on p53 status; VER-150548 did not potentiate the growth inhibitory activity of any of these agents in p53 wild-type HCT116 cells.

Fragment screening and structure guided drug design identified VER-150548 as a novel, potent small molecule inhibitor of Chk and Aurora kinases. In unperturbed human carcinoma cell lines, VER-150548 induced reduplication and inhibited Histone H3 phosphorylation on serine 10, a phenotype consistent with Aurora kinase inhibition in cells. In cells treated with a variety of DNA damaging agents, VER-150548 abrogated both S-phase and G2/M-phase arrest induced by these agents. This abrogation of cell cycle arrest was coupled with the potentiation of cell killing by gemcitabine, camptothecin, cisplatin and doxorubicin in p53 defective but not proficient tumor cells. As with other Chk1 inhibitors such as AZD7762 and PF-477736, the greatest potentiation was observed with gemcitabine [6], [7]. In this case, not only did VER-150548 potentiate the growth inhibitory effect of gemcitabine but increased the fraction of cells killed by this antimetabolite. This increased cell killing was accompanied with an increase in pH2AX (Ser139) levels and suggests that this elevated cytotoxicity is due to greater levels of DNA damage following checkpoint abrogation. The additional stimuli of DNA damage resulted in a cellular phenotype consistent with Chk1 inhibition that was not repressed by activity against the Aurora kinases. Aurora kinase activity would therefore appear dispensable for DNA damage checkpoint abrogation and subsequent potentiation of cytotoxic chemotherapy. Conversely, inhibition of Aurora kinases does not activate a Chk1 dependent DNA damage response and Chk1 activity is not necessary for inducing polyploidy following Aurora inhibition. Checkpoint inhibition is accepted to result in a lethal mitosis due to cells attempting to undertake cell division with extensive chromosomal damage. Since Aurora kinase inhibition prevents the successful conclusion of cytokinesis and cell division, completion of mitosis is not necessary for mitotic catastrophe in cells carrying extensive DNA damage.

Following treatment with a DNA damaging agent, VER-150548 appeared no longer able to induce reduplication and polyploidy in p53 proficient or deficient human carcinoma cells. Treatment with camptothecin or cisplatin plus VER-150548 resulted in the identification of a small fraction (around 10–15%) of cells with a DNA content between 4 and 7N. A closer microscopic analysis of these cells indicated a high number of cells with an aberrant nuclear morphology that is highly suggestive of chromosomal abnormalities and damage. Therefore it is not clear if these cells have escaped mitotic catastrophe, bypassed cytokinesis and attempted S-phase with an incomplete complement of chromosomes or have undergone asymmetrical cell division. A similar phenotype was also observed when camptothecin or cisplatin treated cells were subsequently exposed to a combination of the Chk1 inhibitor PF-477736 and the Aurora inhibitor VX680. The generation of this sub-population of cells with a DNA content between 4 and 7N was dependent on the presence of DNA damage and inhibition of Chk1 kinase, and increased when Aurora kinases were also inhibited. These results are consistent with a small sub-population of cells that have escaped mitotic catastrophe, failed cytokinesis due to Aurora kinase inhibition and attempted S-phase with an incomplete complement of chromosomes. Attempting to replicate extensively damaged DNA in this subsequent S-phase results in further cell death.

Inhibiting Chk1 and Aurora kinases in the presence of DNA damage resulted in a cellular response predominated by the Chk1 inhibitory activity of VER-150548. Why do cells fail to undergo reduplication following treatment with the combination of DNA damaging cytotoxic chemotherapy and our novel kinase inhibitor? We would like to suggest that the temporal arrangement of these two signaling pathways and the timing of response are critical to understanding the cellular phenotype observed. In cells harboring large quantities of potentially lethal DNA damage following treatment with a cytotoxic chemotherapeutic agent, inhibition of the Chk1 kinase relieves cell cycle arrest allowing these cells to enter mitosis. The large quantity of DNA damage sustained by these cells due to checkpoint abrogation results in mitotic catastrophe and subsequent cellular death from this mitosis. This occurs prior to Aurora kinase inhibition, cytokinesis failure and subsequent reduplication. The small fraction of cells escaping this lethal mitotic event will fail cytokinesis due to Aurora kinase inhibition and attempt DNA replication with heavily damaged DNA. This is again likely to be highly lethal. An alternative explanation for the absence of DNA reduplication in the presence of a DNA damaging drug could be that the DNA damage inflicted by the cytotoxic chemotherapeutic drugs inhibits DNA synthesis preventing the subsequent full re-replication of the genome. This would result in cell cycle arrest at this subsequent S-phase. Since the checkpoint kinase Chk1 will still be inhibited by VER-150548, this S-phase arrest would need to occur via a Chk1 independent checkpoint. Our data is much more consistent with the induction of cell death as observed by the massive increase in cells with a sub-G1 DNA content prior to DNA re-replication rather than inhibition of DNA synthesis. Therefore in cells harboring large amounts of potentially lethal DNA damage, inhibition of Chk1 results in cellular death prior to Aurora kinase inhibition thereby preventing DNA reduplication and polyploidy. The temporal arrangement of these two signaling pathways thereby defines why the Chk1 cellular phenotype predominates over the Aurora phenotype in cells treated with cytotoxic chemotherapeutic agents.

In summary, we have identified a relatively non-specific small molecule inhibitor of Chk and Aurora kinases. In unperturbed cells, the Aurora phenotype predominated suggesting that Aurora B is a relatively ‘easy’ kinase to inhibit with the cellular EC_50_ approximating that of the 120 hour GI_50_. At lower doses and in the presence of a DNA damaging agent, the molecule behaves as a Chk1 inhibitor. The temporal arrangement and time to effect of these two signalling pathways thereby determines the signalling network and therefore the cellular phenotype that predominates.

## Methods

### *In vitro* Kinase Assays

Kinase assays were performed as previously described [27]. Details of the assay conditions for each kinase are described in Table 2.

**Table 2:** *In vitro* kinase assay conditions.

	Kinase					
	Aurora A	Aurora B	CDK1/CycB	CDK2/CycA	Chk1	Chk2
**Supplier**	Invitrogen	Invitrogen	Invitrogen	Vernalis	Invitrogen	Invitrogen
**Cat No.**	PV3612	PV3970	PV3292		PV3040	PV3367
**[Enzyme] (nM)**	8	15	52	57	12.5	5
**Cofactors**		0.02 µM INCENP				
**[ATP] (µM)**	10	25	100	100	100	100
**Peptide**	Kemptide	PAKtide	Histone H1	CDKtide	CHKtide	CHKtide
**Sequence**	LRRASLG	RRRLSFAEP	Millipore 14-155	HATTPKKKRK	KKKVSRSGLYRSPSMPENLNRPR	KKKVSRSGLYRSPSMPENLNRPR
**Incubation time (mins)**	30	60	40	40	40	40

### Cell Culture and Cytotoxicity Assay

All cells were obtained from the American Type Culture Collection and cultured in DMEM containing 10% FCS (Invitrogen). The cytotoxicity of VER-150548 was determined following exposure of cells in 96 well plates to a 10-point titration for 72 or 120 hours. Cell proliferation was determined using a CellTiter-Glo® Luminescent Cell Viability Assay kit (Promega).

### Mitotic Index Assay

HT29 cells were seeded in 96 well plates and treated with 25 nM gemcitabine for 16 hours to induce S-phase arrest. Increasing concentrations of VER-150548 were added in the presence of 0.5 µM nocodazole for a further 24 hours before fixing cells with 4% formaldehyde. Mitotic index was determined following cell staining with a phospho-H3 polyclonal antibody and DAPI (Invitrogen). Mitotic cells were scored with a Zeiss Axiovert microscope.

### Antibodies and Western Blotting

Antibodies against the following proteins/epitopes were obtained from the indicated supplier: MPM-2 (ab14581) from Abcam; pHistone H3 (Ser10, 32219) from Millipore; and pPP1α (Thr320, #2581), pChk1 (Ser345, #2341), pCdc2 (Tyr15, #9111) and pH2AX (Ser139, #2577) from Cell Signaling Technologies. Treated and untreated cells were washed once with PBS and lysed in 50 mM Tris-pH6.8, 2% SDS, protease inhibitor cocktail (Roche) and boiled for 5 minutes. Protein concentration was determined using BCA kit (Pierce). Equal amounts of lysate were separated by SDS-PAGE and western blot analysis conducted using the antibodies indicated above.

### Flow Cytometry

HT29 cells were seeded in 6-well plates and subsequently treated with the indicated concentrations of cytotoxic agent or VER-150548 for 8–48 hours. All cells were harvested, fixed in 70% ethanol and stained with propidium iodide/RNase A. Cell cycle profiles were examined by flow cytometry using a FACSArray cytometer (BD) and FACSDiva software (BD).

### Potentiation Assays

HT29 cells were seeded in 96-well plates and treated with a 10-point titration of gemcitabine, camptothecin, cisplatin or doxorubicin in the presence of a fixed concentration of VER-150548 for 72 hours. Cell proliferation was determined using a CellTiter 96 AQ_ueous_ One Solution Cell Proliferation Assay (MTS, Promega).

### Accession codes

3NLB

## Supporting Information

Figure S1 Chemical structure of VER-150548.
(0.04 MB TIF)

Figure S2 VER-150548 does not abrogate gemcitabine or camptothecin induced cell cycle arrest in p53 proficient HCT116 cells. HCT116 cells were mock treated with DMSO or exposed to gemcitabine or camptothecin or for 16 hours followed by DMSO or VER-150548 (200 nM) for a further 24 or 48 hours. Fixed cells were analyzed by flow cytometry.
(0.24 MB TIF)

## References

[1] Zhou BB, Elledge SJ (2000). The DNA damage response: putting checkpoints in perspective.. Nature.

[2] Bucher N, Britten CD (2008). G2 checkpoint abrogation and checkpoint kinase-1 targeting in the treatment of cancer.. Br J Cancer.

[3] O'Connor MJ, Martin NM, Smith GC (2007). Targeted cancer therapies based on the inhibition of DNA strand break repair.. Oncogene.

[4] Bartek J, Lukas J (2003). Chk1 and Chk2 kinases in checkpoint control and cancer.. Cancer Cell.

[5] Liu Q, Guntuku S, Cui XS, Matsuoka S, Cortez D (2000). Chk1 is an essential kinase that is regulated by Atr and required for the G(2)/M DNA damage checkpoint.. Genes Dev.

[6] Zabludoff SD, Deng C, Grondine MR, Sheehy AM, Ashwell S (2008). AZD7762, a novel checkpoint kinase inhibitor, drives checkpoint abrogation and potentiates DNA-targeted therapies.. Mol Cancer Ther.

[7] Blasina A, Hallin J, Chen E, Arango ME, Kraynov E (2008). Breaching the DNA damage checkpoint via PF-00477736, a novel small-molecule inhibitor of checkpoint kinase 1.. Mol Cancer Ther.

[8] Ashwell S, Janetka JW, Zabludoff S (2008). Keeping checkpoint kinases in line: new selective inhibitors in clinical trials.. Expert Opin Investig Drugs.

[9] Matthews DJ, Yakes FM, Chen J, Tadano M, Bornheim L (2007). Pharmacological abrogation of S-phase checkpoint enhances the anti-tumor activity of gemcitabine in vivo.. Cell Cycle.

[10] Zachos G, Black EJ, Walker M, Scott MT, Vagnarelli P (2007). Chk1 is required for spindle checkpoint function.. Dev Cell.

[11] Zhang C, Yan Z, Painter CL, Zhang Q, Chen E (2009). PF-00477736 mediates checkpoint kinase 1 signaling pathway and potentiates docetaxel-induced efficacy in xenografts.. Clin Cancer Res.

[12] Kops GJ, Weaver BA, Cleveland DW (2005). On the road to cancer: aneuploidy and the mitotic checkpoint.. Nat Rev Cancer.

[13] Bekier ME, Fischbach R, Lee J, Taylor WR (2009). Length of mitotic arrest induced by microtubule-stabilizing drugs determines cell death after mitotic exit.. Mol Cancer Ther.

[14] Marumoto T, Zhang D, Saya H (2005). Aurora-A - a guardian of poles.. Nat Rev Cancer.

[15] Fu J, Bian M, Jiang Q, Zhang C (2007). Roles of Aurora kinases in mitosis and tumorigenesis.. Mol Cancer Res.

[16] Vader G, Lens SM (2008). The Aurora kinase family in cell division and cancer.. Biochim Biophys Acta.

[17] Carvajal RD, Tse A, Schwartz GK (2006). Aurora kinases: new targets for cancer therapy.. Clin Cancer Res.

[18] Lapenna S, Giordano A (2009). Cell cycle kinases as therapeutic targets for cancer.. Nat Rev Drug Discov.

[19] Cheung CH, Coumar MS, Hsieh HP, Chang JY (2009). Aurora kinase inhibitors in preclinical and clinical testing.. Expert Opin Investig Drugs.

[20] Girdler F, Gascoigne KE, Eyers PA, Hartmuth S, Crafter C (2006). Validating Aurora B as an anti-cancer drug target.. J Cell Sci.

[21] VanderPorten EC, Taverna P, Hogan JN, Ballinger MD, Flanagan WM (2009). The Aurora kinase inhibitor SNS-314 shows broad therapeutic potential with chemotherapeutics and synergy with microtubule-targeted agents in a colon carcinoma model.. Mol Cancer Ther.

[22] Scharer CD, Laycock N, Osunkoya AO, Logani S, McDonald JF (2008). Aurora kinase inhibitors synergize with paclitaxel to induce apoptosis in ovarian cancer cells.. J Transl Med.

[23] Ditchfield C, Johnson VL, Tighe A, Ellston R, Haworth C (2003). Aurora B couples chromosome alignment with anaphase by targeting BubR1, Mad2, and Cenp-E to kinetochores.. J Cell Biol.

[24] Marumoto T, Honda S, Hara T, Nitta M, Hirota T (2003). Aurora-A kinase maintains the fidelity of early and late mitotic events in HeLa cells.. J Biol Chem.

[25] Bebbington D, Binch H, Charrier JD, Everitt S, Fraysse D (2009). The discovery of the potent aurora inhibitor MK-0457 (VX-680).. Bioorg Med Chem Lett.

[26] Pascreau G, Arlot-Bonnemains Y, Prigent C (2003). Phosphorylation of histone and histone-like proteins by aurora kinases during mitosis.. Prog Cell Cycle Res.

[27] Foloppe N, Fisher LM, Howes R, Kierstan P, Potter A (2005). Structure-based design of novel Chk1 inhibitors: insights into hydrogen bonding and protein-ligand affinity.. J Med Chem.

