# Supplementary figures and images for: Context-Dependent Cell Cycle Checkpoint Abrogation by a Novel Kinase Inhibitor
Source: PLoS One. 2010 Oct 18;5(10):e13123. doi: 10.1371/journal.pone.0013123 (PMC2956624; doi:10.1371/journal.pone.0013123)

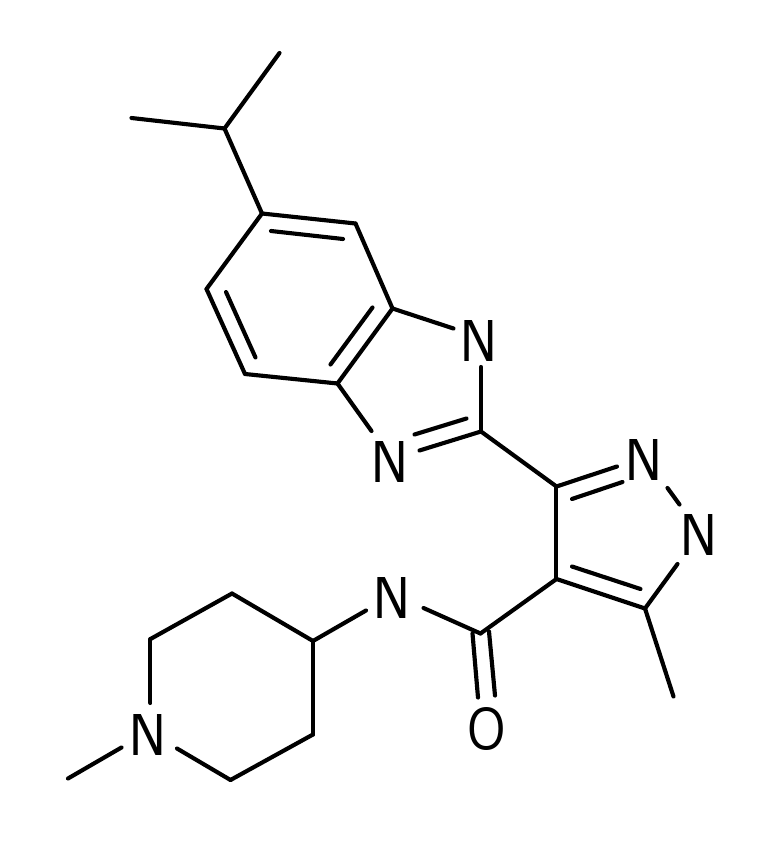

Supplement: Figure S1 — Chemical structure of VER-150548. (0.04 MB TIF) [file pone.0013123.s001.tif]

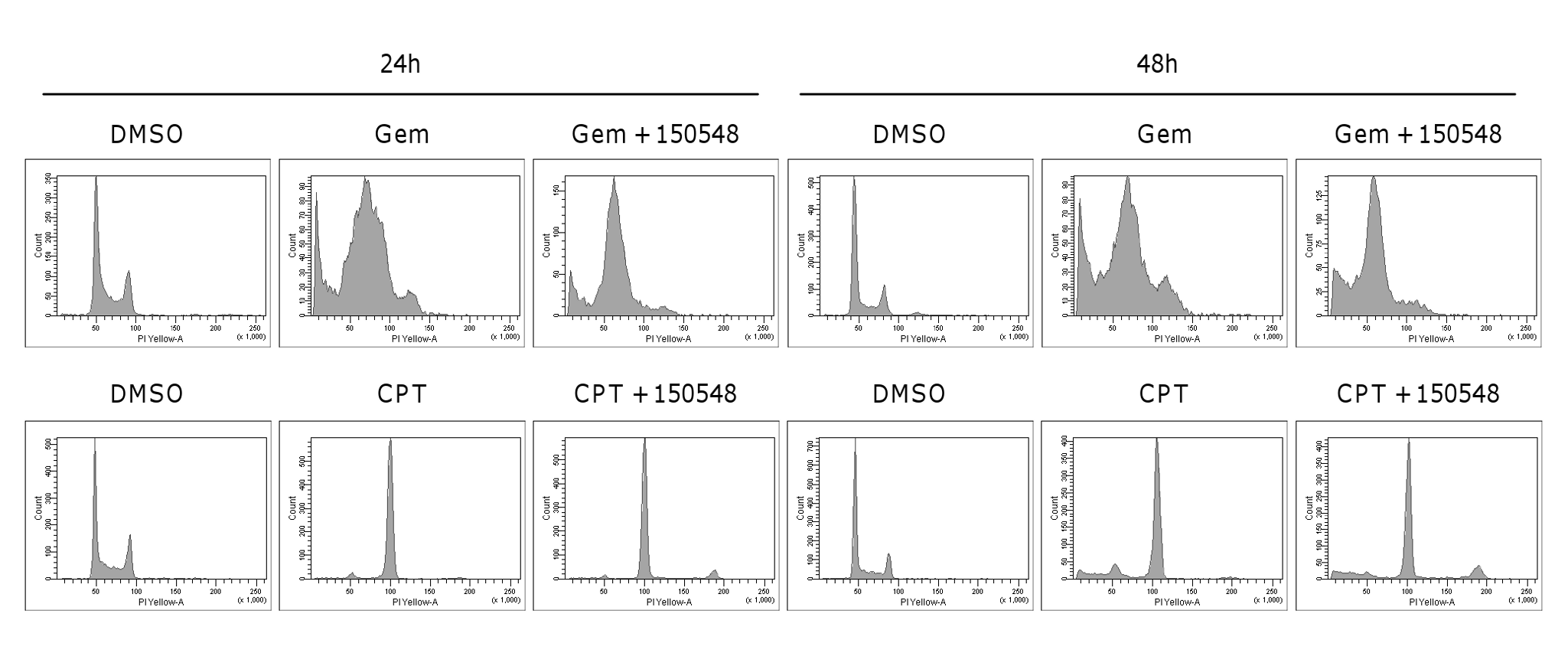

Supplement: Figure S2 — VER-150548 does not abrogate gemcitabine or camptothecin induced cell cycle arrest in p53 proficient HCT116 cells. HCT116 cells were mock treated with DMSO or exposed to gemcitabine or camptothecin or for 16 hours followed by DMSO or VER-150548 (200 nM) for a further 24 or 48 hours. Fixed cells were analyzed by flow cytometry. (0.24 MB TIF) [file pone.0013123.s002.tif]
